# Piperine regulates UCP1 through the AMPK pathway by generating intracellular lactate production in muscle cells

**DOI:** 10.1038/srep41066

**Published:** 2017-01-24

**Authors:** Nami Kim, Miso Nam, Mi Sun Kang, Jung Ok Lee, Yong Woo Lee, Geum-Sook Hwang, Hyeon Soo Kim

**Affiliations:** 1 Integrated Metabolomics Research Group, Western Seoul Center, Korea Basic Science Institute, Seoul 120-140, Republic of Korea; 2 Department of Anatomy, Korea University College of Medicine, Seoul 136-701, Korea; 3 Chemistry & Nanoscience, Ewha Womans University, Seoul 120-750, Republic of Korea

## Abstract

This study characterizes the human metabolic response to piperine, a curcumin extract, and the details of its underlying molecular mechanism. Using ^1^H-NMR-based metabolome analysis, we showed the metabolic effect of piperine on skeletal muscle and found that piperine increased the level of intracellular lactate, an important metabolic intermediate that controls expression of several genes involved in mitochondrial activity. Piperine also induced the phosphorylation of AMP-activated protein kinase (AMPK) and its downstream target, acetyl-CoA carboxylase (ACC), while additionally stimulating glucose uptake in an AMPK dependent manner. Piperine also stimulates the p38 mitogen-activated protein kinase (p38 MAPK), an effect that was reversed by pretreatment with compound C, an AMPK inhibitor. Inhibition of p38 MAPK resulted in no piperine-induced glucose uptake. Increased level of lactate resulted in increased expression of mitochondrial uncoupling protein 1 (UCP1), which regulates energy expenditure, thermogenesis, and fat browning. Knock-down of AMPK blocked piperine-induced UCP1 up-regulation, demonstrating the required role of AMPK in this effect. Taken together, these results suggest that piperine leads to benign metabolic effects by activating the AMPK-p38 MAPK signaling pathway and UCP1 expression by activating intracellular lactate production in skeletal muscle.

Metabolic disorders, obesity, and type 2 diabetes are major and increasingly important health disorders caused, at least in part, by modern lifestyles and habits^1^,^2^. Insulin resistance and impaired glucose and lipid metabolism are key components of type 2 diabetes pathophysiology^3^. AMP-activated protein kinase (AMPK), an energy sensor and master regulator of metabolic homeostasis, acts to increases glucose uptake, fatty acid oxidation, and mitochondrial biogenesis, all of which ameliorate insulin resistance^4^,^5^. AMPK is a heterotrimeric protein composed of α, β, and γ subunits; there are two isoforms each of α (α1 and α2) and β (β1 and β2) and three of γ (γ1, γ2, and γ3)^6^. An increased AMP:ATP ratio activates phosphorylation of Thr172 in the activation loop of the α subunit catalytic domain, enabling AMPK catalytic activity^7^. Although the molecular mechanisms underlying AMPK activation remain largely unknown, it is thought to mediate glucose uptake and fatty acid oxidation during exercise by phosphorylating and inhibiting acetyl CoA carboxylase (ACC)^8^,^9^. AMPK activation also induces glucose uptake into fat and muscle cells via the glucose transporter type 4 (Glut4) on the plasma membrane^10^.

Piperine, a curcumin derivative, is an alkaloid found in the seeds of black pepper, and it is commonly used to treat seizures in traditional Chinese medicine^11^,^12^. Various physiological benefits of piperine treatment have been reported, including anti-inflammatory, anti-cancer, anti-oxidant, and hepatoprotective effects^13^,^14^,^15^. Piperine has been shown to reverse hepatic steatosis and insulin resistance in mice by regulating AMPK, while also reducing obesity by regulating lipid metabolism^16^,^17^. Our group previously reported that curcumin and its analogue, dibenzoylmethane, demonstrate multiple biological activities, including increased glucose uptake by activating AMPK in skeletal muscle^18^,^19^. Despite these demonstrated positive metabolic changes, the mechanisms underlying piperine’s effects in skeletal muscle remains largely unknown.

In order to more fully characterize the metabolic effect of piperine, we investigated its role in skeletal muscle when used as treatment for several metabolic disorders. Our results show that piperine induces glucose uptake in skeletal muscle cells by activating the AMPK and p38 mitogen-activated protein kinases (MAPK) signaling pathways. Measurements by ^1^H-NMR also suggest that piperine increases intracellular lactate level, which is known to induce AMPK activation and UCP1 expression and thereby positively regulate thermogenesis, energy expenditure, and protection against oxidative stress^20^. We thus conclude that piperine regulates metabolic processes by activating the AMPK signaling pathway in a manner that makes it a strong candidate for clinical metabolic therapy.

## Results

### Piperine-treated C2C12 myoblast extracts show distinct metabolite levels

**Figure 1:**
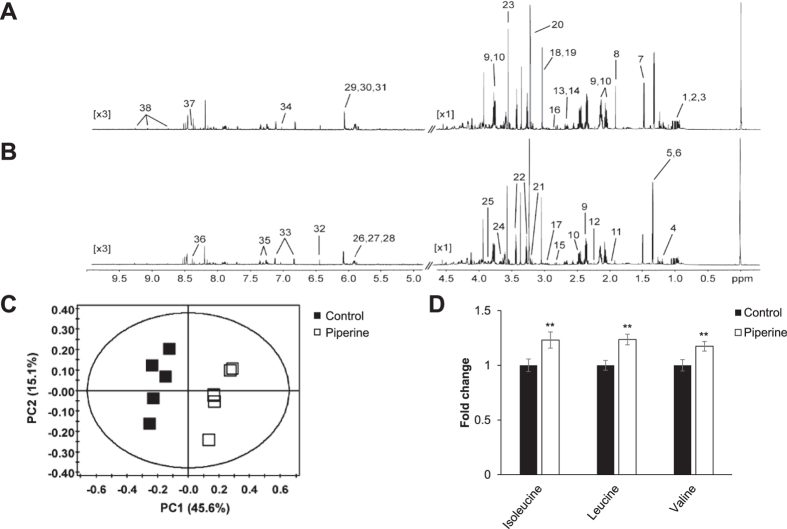
Piperine affects cell energy metabolism. C2C12 cells were stimulated with 30 μM piperine for 3 h. The cell metabolites were extracted with MeOH/water/CHCl_3_, and NMR-based metabolic profiling analysis was conducted. The representative ^1^H-NMR spectra of cell extracts from **(A)** control and **(B)** piperine are shown. (1, Valine; 2, Leucine; 3, Isoleucine; 4, Ethanol; 5, Lactate; 6, Threonine; 7, Alanine; 8, Acetate; 9, Glutamate; 10, Glutamine; 11, Proline; 12, Acetone; 13, Methionine; 14, Malate; 15, Aspartate; 16, Asparagine; 17, Glutathione; 18, Creatine; 19, Creatine phosphate; 20, O-phosphocholine; 21, Choline; 22, Taurine; 23, Glycine; 24, myo-Inositol; 25, Serine; 26, UDP-glucose; 27, UDP-galactose; 28, UDP-glucuronate; 29, ADP; 30, AMP; 31, ATP; 32, Fumarate; 33, Tyrosine; 34, Histidine; 35, Phenylalanine; 36, Formate; 37, NADH; and 38, NAD^+^). **(C)** PCA score plot for ^1^H-NMR spectra of cell metabolite extract levels for metabolome analysis. **(D)** Levels of BCAAs isoleucine, leucine, and valine in cell metabolite extracts. **P < 0.01 compared with the untreated cells. Results from three independently replicated experiments.

To determine whether piperine induces metabolic effects in C2C12 cells, we performed ^1^H-NMR-based metabolome analysis. Representative 800-MHz ^1^H-NMR spectra differed in the absence (Fig. 1A) and presence (Fig. 1B) of treatment with 30 μM piperine for 3 h. Comparison of 38 metabolite concentrations measured in cell extracts from five controls and five piperine-treated C2C12 samples revealed substantial differences. We quantified these differences using principal component analysis (PCA) (Fig. 1C), which shows a clear separation between piperine-treated and untreated cells (PCA: R^2^X and Q^2^X values of 0.607 and 0.341, respectively). Cell metabolite extracts from piperine-treated groups contained significantly higher levels of branched-chain amino acids (BCAAs) isoleucine, leucine, and valine (Fig. 1D). BCAAs are potential regulators of glucose, leptin, cell signaling, adiposity, and body weight^21^, and increases in their concentrations have been associated with improvement in insulin resistance^22^. These results suggest that piperine induces numerous changes in metabolite levels, at least some of which are likely to affect energy metabolism, particularly through BCAA activation.

### Piperine upregulates mitochondrial oxygen consumption by increasing the AMP:ATP ratio in C2C12 myoblasts

**Figure 2:**
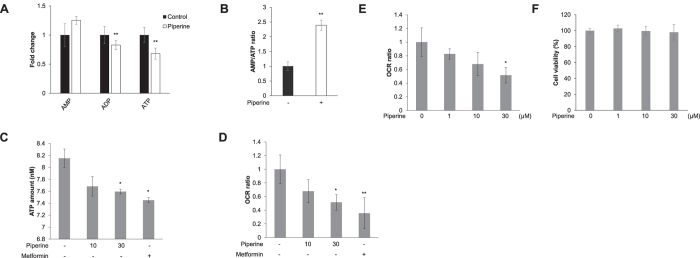
Piperine increases AMPK phosphorylation by mitochondrial oxygen consumption. **(A)** ^1^H-NMR-based metabolic profiling analysis shows the intensity of adenosine phosphates ATP, ADP, and AMP in piperine-treated or untreated cell metabolite extracts. **(B)** AMP:ATP ratio from ^1^H-NMR spectral intensities. **(C)** C2C12 cells were treated with 30 μM piperine or 10 mM metformin for 24 h. The cells were lysed with ATP assay buffer, and the amount of ATP was evaluated by colorimetric absorbance assay. Metformin was used as a positive control. **(D)** C2C12 cells were treated with 30 μM piperine or 10 mM metformin for 24 h. Mitochondrial oxygen consumption rate (OCR) was measured using an XF24 analyzer. Metformin was used as a positive control. **(E)** C2C12 cells were treated with indicated doses of piperine for 24 h. Mitochondrial oxygen consumption rate (OCR) was measured using an XF24 analyzer. *P < 0.05 compared with the untreated cells. Results from three independently replicated experiments. **(F)** C2C12 cells were treated with indicated doses of piperine for 24 h. Cell viability was analyzed with MTT assay. Results from three independently replicated experiments. *P < 0.05, **P < 0.01 compared with the untreated cells. Results from three independently replicated experiments. Cropped images of full-length blots are shown.

Adenosine phosphate (ATP, ADP, and AMP) levels were quantified by ^1^H-NMR spectra and showed that piperine-treatment increased the level of AMP but decreased those of ADP and ATP (Fig. 2A), thereby markedly increasing the AMP:ATP ratio (Fig. 2B). To determine whether piperine exerted energy metabolic effects in C2C12, we also quantified the intracellular level of ATP via colorimetric assay. Administration of piperine or metformin for 24 h decreased the amount of ATP compared to controls (Fig. 2C). We performed extracellular flux analysis in order to assess mitochondrial respiration in C2C12 cells. Figure 2D shows that treatment with piperine or metformin for 24 h significantly decreased the basal oxygen consumption rate (OCR). Piperine decreased the basal oxygen consumption rate in a dose-dependent manner (Fig. 2E). There was no cytotoxic effect up to 30 μM of piperine (Fig. 2F). Together, these results indicate that piperine-induced elevation of the AMP:ATP ratio through ATP consumption blocks mitochondrial respiration in C2C12 cells.

### Piperine increases AMPKα phosphorylation in C2C12 myoblasts in a dose- and time-dependent manner

**Figure 3:**
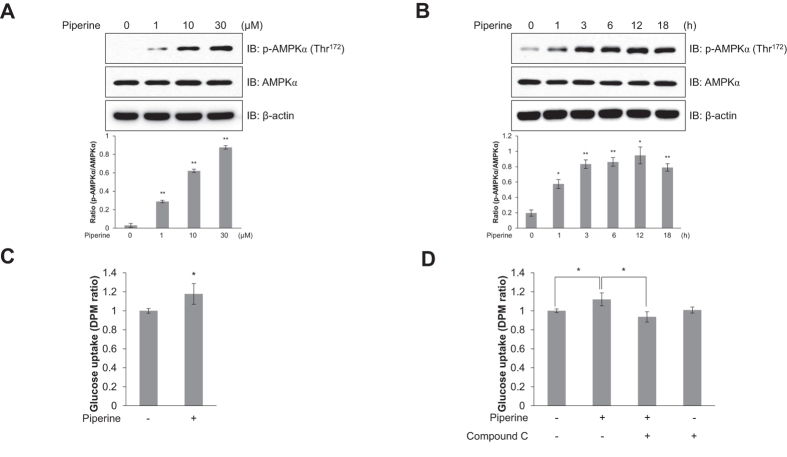
Piperine stimulates glucose uptake through AMPK. **(A)** C2C12 cells were treated for 3 h with various concentrations of piperine or **(B)** with 30 μM piperine for the duration of time indicated. Cells were then lysed, and AMPKα phosphorylation was quantified by Western blot using antibodies specific to the phosphorylated protein. The levels of total AMPKα and β-actin were also measured as controls for protein loading. **(C)** L6 myotubes were differentiated for 7 days and treated with 30 μM piperine for 18 h before 2-deoxy-d[H^3^]-glucose (2-DG) uptake was measured. **(D)** Differentiated L6 myotubes were treated with 30 μM piperine for 18 h in either the presence or absence of compound C (5 μM). *P < 0.05, **P < 0.01 compared with the untreated cells. Results from three independently replicated experiments. Blots were displayed in cropped format. Cropped images of full-length blots are shown.

AMPK is activated by an increased cellular AMP:ATP ratio, which occurs during cellular stress in mammalian cells. Recent research demonstrated that AMPK is also activated by increases in ADP. Although best known for its effects on metabolism, AMPK has many other functions, including regulation of mitochondrial respiration and disposal, autophagy, cell polarity, and cell growth and proliferation^23^. To identify the mechanism underlying the metabolic effects of piperine in C2C12 myoblasts, we evaluated the phosphorylation of AMPKα, which is known to activate AMPK catalytic activity, and is a key mechanism of glucose uptake. Piperine induced phosphorylation of AMPKα in a dose- and time-dependent manner in C2C12 myoblasts (Fig. 3A and B). High level of AMPKα phosphorylation was observed at 30 μM piperine and reached a maximum after 12 h. Piperine also stimulated glucose uptake in L6 myotubes (Fig. 3C). However, this effect was blocked by 5 μM compound C, an AMPK inhibitor (Fig. 3D). These results indicate that piperine increases glucose uptake through AMPKα phosphorylation in C2C12 myoblasts.

### Piperine activates the p38 MAPK pathway through AMPKα in C2C12 myoblasts

**Figure 4:**
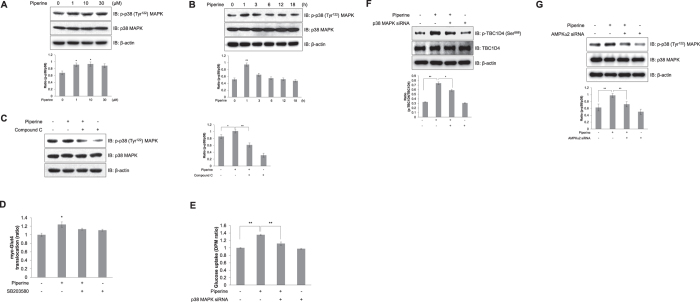
Piperine activates the p38 MAPK pathway in an AMPK-dependent manner. **(A)** C2C12 cells were treated with different concentrations of piperine for 3 h, **(B)** with 30 μM piperine for the duration of time indicated, or **(C)** with 30 μM piperine for 3 h in the presence of 5 μM compound C. The cells were lysed, and the phosphorylation of p38 MAPK was quantified by Western blot using antibodies specific for the phosphorylated protein. The levels of total p38 MAPK and β-actin were also measured as controls for protein loading. **(D)** Myoblasts stably expressing L6-Glut4-myc were differentiated for 7 days, pretreated with SB203580 (5 μM) for 30 min, and then incubated with piperine for 3 h. Cell surface expression of Myc-Glut4 was quantified using an antibody-coupled colorimetric absorbance assay. **(E)** Differentiated L6 myotubes were transiently transfected with p38 MAPK siRNA (100 nM) for 48 hours and then treated with 30 μM piperine before 2-deoxy-d[H^3^]-glucose (2-DG) uptake was measured. **(F)** C2C12 cells were transfected with p38 MAPK siRNA (100 nM) for 2 days and then treated with piperine (30 μM) for 1 h. The cells were lysed, and the phosphorylation of TBC1D4 was quantified by Western blot analysis. The level of TBC1D4 was measured as a control for protein loading. Results from three independently replicated experiments. **(G)** C2C12 cells were transfected with AMPKα2 siRNA (100 nM) for 2 days and then treated with piperine (30 μM) for 1 h. The cells were lysed, and the phosphorylation of p38 MAPK was quantified by Western blot analysis. The level of p38 MAPK was measured as a control for protein loading. Results from three independently replicated experiments. *P < 0.05, **P < 0.01 compared with the untreated cells. Results from three independently replicated experiments. Blots were displayed in cropped format. Cropped images of full-length blots are shown.

As a result of AMPKα activation, p38 MAPK activity was markedly increased by piperine treatment. Piperine induced phosphorylation of p38 MAPK in a dose- and time dependent manner (Fig. 4A and B). In contrast, p38 MAPK phosphorylation was attenuated in C2C12 myoblasts pretreated with 5 μM compound C (Fig. 4C), demonstrating the essential role of AMPK in this process. p38 MAPK regulates glucose transporter expression and glucose uptake^24^. Piperine-treated cells showed increased level of Myc-Glut4 on the plasma membrane, indicating that piperine induces Glut4 translocation from the cytosol to the plasma membrane (Fig. 4D). The addition of SB203580 (5 μM), a p38 MAPK inhibitor, significantly inhibited piperine-induced Glut4 translocation (Fig. 4D). Knock-down of p38 MAPK blocked piperine-mediated glucose uptake (Fig. 4E). Target of AMPK, TBC1D4 phosphorylation was also suppressed by p38 MAPK knock-down (Fig. 4F). Knock-down of AMPKα2 blocked piperine-induced p38 MAPK phosphorylation (Fig. 4G). These results suggest that piperine stimulates glucose uptake by activating Glut4 translocation through the AMPKα-p38 MAPK signaling pathway.

### Piperine positively regulates Glut4 expression and translocation in an AMPKα2-dependent manner

**Figure 5:**
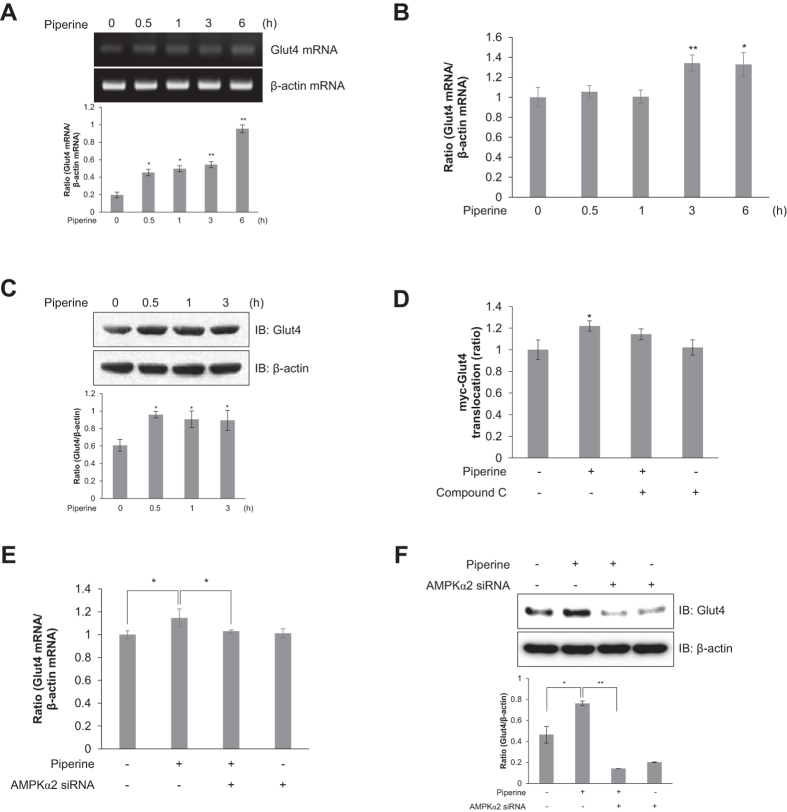
Piperine induces Glut4 translocation in an AMPK-dependent manner. **(A)** Total mRNA was extracted from piperine-treated C2C12 cells, and RT-PCR was performed using specific Glut4 and β-actin primers. PCR products were separated on 1% agarose gels and visualized under ultraviolet light. β-Actin was used as a positive control. **(B)** C2C12 cells were treated with piperine (30 μM) for indicated times. Total mRNA was extracted from each sample, and quantitative RT-PCR was performed using specific primers for Glut4 and β-actin transcripts. **(C)** C2C12 cells were stimulated for duration of time indicated with 30 μM piperine. The cells were lysed, and the expression of Glut4 and β-actin was quantified using Western blot analysis. The levels of β-actin were also measured as a control for protein loading. **(D)** Stably expressed L6-Myc-Glut4 myoblasts were pretreated with compound C (5 μM) and then incubated with piperine for 3 h. Cell surface expression of Myc-Glut4 was measured using an antibody-coupled colorimetric absorbance assay. **(E)** C2C12 cells were transfected with AMPKα2 siRNA (100 nM) for 2 days and then treated with piperine (30 μM) for 3 h. Total mRNA was extracted from each sample, and quantitative RT-PCR analysis was performed using specific primers for Glut4 and β-actin transcripts. **(F)** C2C12 cells were transfected with AMPKα2 siRNA (100 nM) for 2 days and then treated with piperine (30 μM) for 3 h. The cells were lysed, and the expression of Glut4 and β-actin was quantified by Western blot analysis. The level of β-actin was measured as a control for protein loading. *P < 0.05, **P < 0.01 compared with the basal condition. Results from three independently replicated experiments. Blots were displayed in cropped format.

Glut4 is the principal transporter mediating glucose uptake and plays a key role in regulating whole-body glucose homeostasis^25^. Piperine treatment increased the Glut4 mRNA and relative mRNA levels in a time-dependent manner in C2C12 myoblasts (Fig. 5A and B). Furthermore, piperine treatment activated Glut4 protein expression in a time-dependent manner (Fig. 5C). Pretreatment with compound C resulted in no significant piperine-induced translocation of Glut4 to the plasma membrane (Fig. 5D). This indicates that AMPK is a critical component of Glut4-mediated glucose uptake. To further test this effect, siRNA targeting AMPKα2 was used to knockdown the piperine-activated AMPKα2 gene expression. As shown in Fig. 5E, while piperine increased the relative Glut4 mRNA level compared with controls, AMPKα2 siRNA significantly reversed this effect. Similarly, piperine-stimulated Glut4 protein expression was reduced by siRNA knockdown of AMPKα2 (Fig. 5F). These results suggest that piperine stimulates Glut4 expression by increasing AMPKα2 expression level in C2C12 myoblasts.

### Piperine increases AMPKα phosphorylation and glucose uptake in primary cultured myoblasts

**Figure 6:**
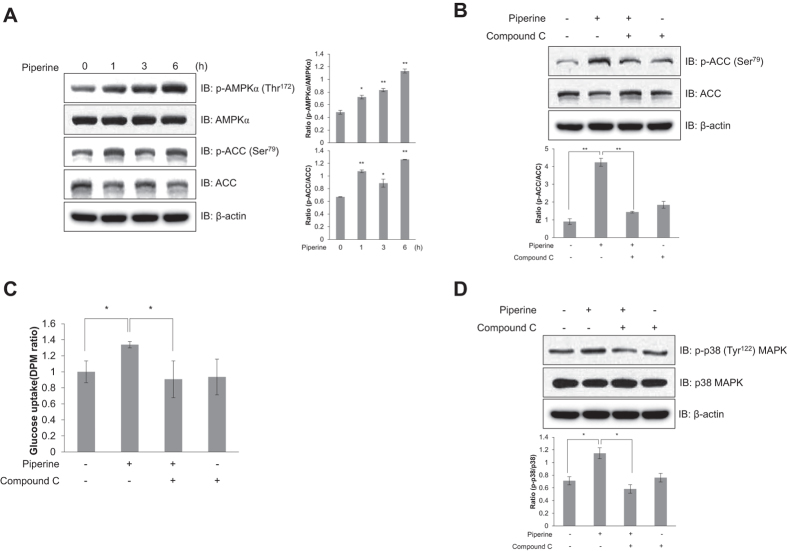
Piperine activates AMPK and stimulates glucose uptake in primary cultured myoblasts. **(A)** Primary cultured myoblasts were stimulated with 30 μM piperine for the indicated times. Cell lysates were analyzed by Western blot analysis with antibodies specific to phosphorylated ACC and phosphorylated AMPKα. Blotting with antibodies specific to non-phosphorylated ACC, AMPKα, and β-actin served as controls. **(B)** Primary cultured myoblasts were treated with compound C (5 μM) for 30 min and then stimulated with piperine (30 μM) for 3 h. Cell lysates were used for Western blot analysis with an antibody specific to phosphorylated ACC. Blotting with antibodies specific to non-phosphorylated ACC and β-actin were used as controls. **(C)** Primary myoblasts were differentiated for 3 days. The cells were then treated with compound C for 30 min and then stimulated with piperine (30 μM) for 3 h before 2-DG uptake was assayed. **(D)** Differentiated primary myotubes were pretreated with 5 μM compound C for 30 min and treated with piperine for 3 h. The cells were lysed with lysis buffer, and the expression of phosphorylated p38 MAPK, non-phosphorylated p38 MAPK, and β-actin was quantified by Western blot analysis. The levels of non-phosphorylated p38 MAPK and β-actin were also measured as a control for protein loading. *P < 0.05, **P < 0.01 compared with the untreated cells. Results are from three independently replicated experiments. Blots were displayed in cropped format.

We next investigated the effects of piperine on primary cultured myoblasts. Piperine time-dependently increased phosphorylation of AMPKα in primary cultured myoblasts (Fig. 6A), while also increasing the phosphorylation of ACC, the downstream target of AMPK (Fig. 6A). Inhibition of AMPK by compound C abolished the piperine-induced phosphorylation of ACC (Fig. 6B).

To characterize the functional significance of AMPK, we measured glucose uptake in primary cultured myoblasts. Similar to the *in vitro* results, piperine-treated cells demonstrated increased glucose uptake; however, compound C blocked this effect (Fig. 6C). To confirm the role of AMPK, we quantified the level of p38 MAPK phosphorylation in primary cultured myoblasts pretreated with compound C and showed that piperine treatment no longer had any effect (Fig. 6D). Thus, these results indicate that piperine-induced glucose uptake occurs via the AMPK-p38 MAPK signaling pathway in primary cultured myoblasts.

### Piperine regulates UCP1 expression by inducing intracellular lactate release in C2C12 myoblasts

**Figure 7:**
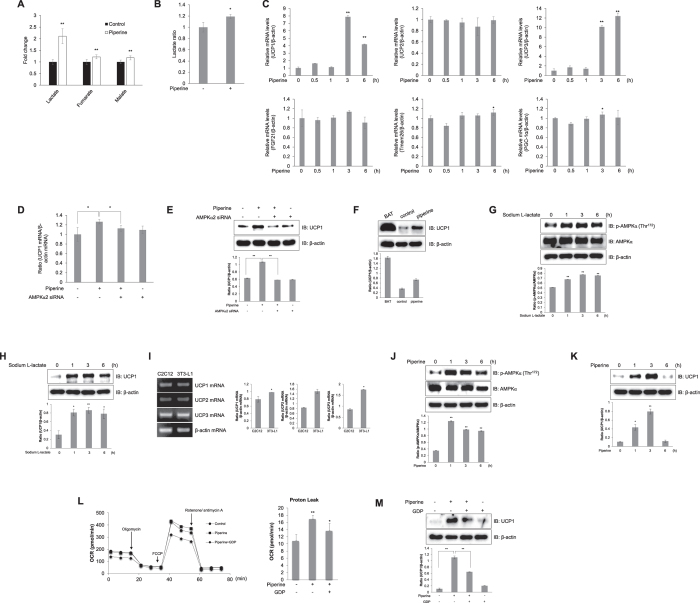
Intracellular lactate concentration mediates piperine induction of UCP1 in an AMPK-dependent manner. **(A)** ^1^H-NMR-based metabolic profiling analysis shows the levels of lactate, fumarate, and malate in piperine-treated or untreated cell metabolite extracts. **(B)** C2C12 cells were treated with 30 μM piperine. Lactate was detected using a lactate colorimetric absorbance assay. **(C)** C2C12 cells were treated with piperine (30 μM). Quantitative RT-PCR analysis was performed with specific primers. β-actin was used as an endogenous control. **(D)** C2C12 cells were transiently transfected with AMPKα2 siRNA (100 nM) and then treated with piperine (30 μM). Quantitative RT-PCR analysis was performed. **(E)** C2C12 cells were transiently transfected with AMPKα2 siRNA (100 nM) and then treated with piperine (30 μM). Expression of UCP1 and β-actin was quantified by Western blot analysis. **(F)** C2C12 cells were treated with 30 μM piperine. BAT of C57BL/6 mouse were obtained from Korea University. The cells and BAT were lysed, and the expression of UCP and β-actin was quantified by Western blot analysis. The level of β-actin was measured as a control for protein loading. **(G)** C2C12 myoblasts were stimulated with sodium-L-lactate (30 mM). Cell lysates were analyzed by Western blot analysis with antibodies. **(H)** C2C12 myoblasts were stimulated with sodium-L-lactate. Cell lysates were analyzed by Western blot analysis. **(I)** Total mRNA was extracted from C2C12 and 3T3-L1 cells, and quantitative RT-PCR was conducted. **(J)** 3T3-L1 pre-adipocytes were stimulated with piperine. Cell lysates were analyzed by Western blot analysis. **(K)** 3T3-L1 pre-adipocytes were stimulated with piperine. Cell lysates were analyzed by Western blot analysis. **(L)** C2C12 cells were pre-treated with 1 mM GDP for 30 min and treated with 30 μM piperine for 24 h. Mitochondrial oxygen consumption rate (OCR) in cells was measured using the XF Seahorse flux analyzer in response to 1 μM oligomycin, 1 μM FCCP, and 0.5 μM rotenone/antimycin A. The proton leak calculated from left figure result. **(M)** C2C12 cells were pre-treated with 1 mM GDP for 30 min and treated with 30 μM piperine for 3 h. Mitochondria lysates were analyzed by Western blot analysis. *P < 0.05, **P < 0.01 compared with the untreated controls. Results are from three independently replicated experiments. Cropped images of full-length blots are shown.

The ^1^H-NMR results showed that piperine increased the levels of lactate, fumarate, and malate, three TCA cycle intermediates (Fig. 7A)^26^. In particular, piperine significantly increased the intracellular lactate level in C2C12 myoblasts (Fig. 7A and B). To examine the mechanism by which piperine links mitochondrial function and lactate level in skeletal muscles, we performed quantitative RT-PCR for several mitochondria-related genes. Among tested genes, piperine increased relative mRNA level of UCP1 and UCP3 (Fig. 7C). To investigate whether the AMPK pathway was involved in UCP1 expression, we used AMPKα2 siRNA. AMPKα2 silencing reduced the effect of piperine on UCP1 relative mRNA (Fig. 7D) and protein levels (Fig. 7E). To confirm the effect of piperine, we performed Western blot analysis with specific UCP1 antibody. The UCP1 expression increased in piperine-treated C2C12 cells (Fig. 7F). The basal level of UCP1 in brown adipose tissue (BAT) was much higher than that of C2C12 cells. Lactate itself increased the phosphorylation of AMPKα (Fig. 7G) and the expression of UCP1 (Fig. 7H). To compare UCP expression, we used 3T3-L1 pre-adipocytes. Three kinds of UCP were expressed both in skeletal muscles and adipocytes (Fig. 7I). Piperine induced phosphorylation of AMPKα in 3T3-L1 cells (Fig. 7J). In addition, piperine increased the expression of UCP1 in this cell (Fig. 7K). To characterize the role of piperine as mitochondria respiration regulator, we examined extracellular mitochondria flux with GDP, an UCP1 inhibitor. Administration of FCCP, a mitochondrial membrane uncoupler, significantly decreased OCR levels in GDP pre-treated C2C12 cells. Representative date show that treatment with piperine increased proton leak but pre-treatment with GDP blocked this effect (Fig. 7L). To confirm the effect on mitochondria respiration, we examined UCP1 expression using isolated mitochondria. Piperine-mediated UCP1 induction was not observed in the presence of GDP (Fig. 7M). These results suggest that piperine induces intracellular lactate release to activate the AMPK signaling pathway and thus involve in mitochondrial respiration.

## Discussion

In this study, we investigate how piperine, an analogue of curcumin, effects metabolism and regulates mitochondria through AMPK signaling activation in skeletal muscles. Recent metabolomics studies show that plasma levels of BCAAs and other essential amino acids are elevated in cells that demonstrate insulin resistance^27^. In addition, a significant increase in BCAA level and the AMP:ATP ratio enhances energy production and catabolic activities, such as fatty acid oxidation and glucose transport, by activating AMPK expression^28^. Treatment with a combination of curcuminoid-piperine has been shown to improve the oxidative and inflammatory states in patients with metabolic syndromes, and piperine also reverses the HFD-induced downregulation of the adiponectin-AMPK pathway, mediating lipogenesis, fatty acid oxidation, and insulin level in mouse liver^16^,^29^. However, the precise mechanism and role of piperine as it relates to levels of BCAAs and AMPK pathway activation in skeletal muscle have not yet been identified. We observed that the mitochondrial oxygen consumption rate was reduced in piperine-treated skeletal muscle cells. The decreased oxygen consumption rate was related to the glycolysis rate. In addition, piperine increased the intracellular AMP:ATP ratio and the level of BCAAs, which promote phosphorylation of AMPK and increase glucose uptake in skeletal muscle cells. Our data suggest that piperine stimulates AMPK activation, which subsequently induces glucoses uptake in skeletal muscle. Glucose downregulation by piperine might be related to the increase in intracellular BCAA level and greater activation of the AMPK signaling pathway.

Over the past decade, numerous studies have reported that AMPK is a highly conserved cellular energy sensor and an important regulator of energy metabolism in response to physiological stimuli such as exercise, stress and hormones^30^,^31^. This is particularly true in the skeletal muscle, where AMPK plays an essential role in maintaining mitochondrial capacity and promoting glucose uptake through muscle contraction, which is critical for insulin sensitivity, fatty acid oxidation, and glycogen synthesis^31^,^32^. Contraction-activated AMPK affects Glut4 translocation and the p38 MAPK pathway downstream of AMPK, which activates glucose uptake^33^,^34^. We used two inhibitors and one siRNA to silence AMPKα2 in skeletal muscle cells, suspecting the AMPK pathway to be critical for the mechanism of piperine-induced effects on metabolism. The AMPK inhibitor compound C downregulated stimulation of p38 MAPK, and the p38 MAPK inhibitor SB203580 inhibited Glut4 translocation. Furthermore, AMPKα2 knockdown abolished the piperine-activated Glut4 expression and glucose uptake. Similarly, our primary culture experiment showed that piperine treatment induced glucose uptake via AMPK-p38 MAPK activation, resulting in greater translocation of Glut4 into the plasma membrane, thereby activating glucose uptake through the AMPK and p38 MAPK signaling pathway in skeletal muscle. In this study, knock down of p38 MAPK blocked piperine-mediated glucose uptake. This result is consistent with the reports that showed the involvement of p38 MAPK in glucose uptake^35^,^36^,^37^. It is reported that p38 MAPK activates PGC-1^38^, a key molecule that regulates mitochondria biogenesis. Collectively, these facts indicate that p38 MAPK might play a critical regulator for mitochondria.

Prior works have identified piperine’s effects on the AMPK pathway, as well as glucose uptake and lipid oxidation, and many aspects of its role in metabolic disease such as diabetes and obesity and its complications have been elucidated. Nevertheless, AMPK activators like piperine are not typically used for clinical treatment of these disorders, despite their pharmacological potential, because the mechanisms of their action remained unknown. By demonstrating a new mechanism by which piperine regulates metabolite levels and fat-browning gene expression through AMPK signaling, this study provides the mechanistic insight necessary to enable further clinical testing with this compound.

We also found that piperine increased TCA cycle intermediate metabolites such as fumarate and malate in skeletal muscle cells, with lactate level being particularly stimulated. Lactate is a glycolytic product that is formed and utilized continuously in diverse cells under fully aerobic conditions, while also being induced by lack of oxygen during skeletal muscle contraction^39^. A recent study demonstrated that lactate could be a useful target for clinical research. Blood lactate is also an important systemic energy source for the human brain and is the main substrate during central nervous system development^40^,^41^. Lactate administration reproduces specific exercise mimetic changes through gluconeogenesis-promoting genes such as peroxisome proliferator-activated receptor-gamma co-activator 1 alpha (PGC-1α) and PGC-1β regulation in the brain and liver^42^. In fact, a previous study reported that the presence of lactate directly affects energy metabolism by modifying bioenergetics fluxes, including p38 MAPK, AMPK, and mammalian target of rapamycin, a downstream target of AMPK^43^. With this work, we demonstrate that piperine increases intracellular lactate and thereby affects AMPK signaling, which is in turn required for mitochondrial respiration under conditions of chronic energy deprivation^44^. Thus, our results elucidate, in detail, the connection between AMPK and mitochondrial respiration-related genes through intracellular lactate stimulation by piperine. In addition, we demonstrated that piperine treatment caused an increase in lactate and increased expression of UCP1 in skeletal muscle C2C12 cells.

UCP1 is a molecular mechanism for heat generation^45^. It is chiefly expressed in brown adipose tissue, where it regulates thermogenesis and energy expenditure while also protecting against oxidative stress^20^. Lactate is also known to control UCP1 expression by inducing browning in human and murine white adipocytes, and UCP1 overexpression improves insulin sensitivity in obesity-resistant rats^46^,^47^. Previous work with rat muscle demonstrated that lactate can drive mitochondrial gene expression and promote fatty acid oxidation, mitochondrial activity, and expression of UCP1^45^. Lactate is released from cells as the end metabolites of fermentation. Lactate is believed to be a waste product of glycolysis. In this study, we found that piperine generated lactate in muscle. Based on this notion, clinical usefulness of piperine should be limited. At the same time, however, it is known that lactate taken up by cells and used to synthesize other metabolites. Further studies are required to demonstrate the effects of piperine in metabolism.

The expenditure of energy through UCP1 was characterized in brown adipose tissue (BAT). UCP2 and UCP3 bear high degree of sequence homology to UCP1. Their role of energy expenditure have not been characterized to the extent of UCP1, because their expression are not confined to BAT. UCP1 expression is not exclusively confined to BAT. The ectopic expression of UCP1 in skeletal muscle was found to have a beneficial effect on glucose metabolism^48^,^49^. We also showed that 3 isozymes of UCP1 were expressed in both skeletal muscle and adipocyte cell (Fig. 7I). These facts indicated that UCP1 may be up-regulated in skeletal muscle by metabolic regulator, like AMPK and may involve in glucose metabolism in skeletal muscle, as well as energy expenditure in BAT. We examined the expression of inflammatory markers, such as TGFα, IL-1β, IL-6, TNFα. The expression levels of these genes were not affected by piperine (Data not shown). These results indicated that the major mechanism of piperine is mediated by UCP1, not by compensatory inflammatory gene regulation.

We previously reported that curcumin stimulated glucose uptake in skeletal muscles^50^. The clinical usefulness of curcumin has been limited due to its low bioavailability caused by poor absorption and faster metabolic alteration. It was reported that piperine enhanced curcumin’s effect not only by reducing curcumin’s metabolic breakdown, but also by increasing the absorption of curcumin in intestine^51^,^52^. Piperine is a structural analogue of curcumin, and its molecular weight is smaller than curcumin. In the point of human application, small molecule is more useful. Therefore, piperine is a promising molecule for the development of diabetes by enhancing curcumin’s beneficial metabolic effect.

The key downstream effector of piperine is AMPK. AMPK is also functional target of metformin, a well-known diabetic drug. Metformin inhibits complex 1 respiratory chain and thus increases ADP/AMP:ATP ratio. It is thought that metformin activates AMPK ADP/AMP:ATP ratio independently. Piperine causes a reduction in mitochondrial respiration and increase AMPK via lactate generation. Therefore, in the point of complexity, it is yet to be delineated the molecular signal network of metformin and piperine. Piperine dramatically inhibited OCR, and at the same time, increased the ratio of AMP/ATP. In addition, piperine increased the UCP1 expression in isolated mitochondria. Collectively, these facts indicated that piperine may work via regulating mitochondrial respiration. Recently, roles of AMPK in brown adipocyte were reported. One paper showed that AMPK in adipocyte was vital for mitochondrial integrity^53^. Lack of AMPK in adipocytes exacerbated insulin resistance and hepatic steatosis. The other article demonstrated that AMPK was essential for the epigenetic control of BAT development^54^. AMPK affected BAT development via changing an important metabolite, alpha-ketoglutarate. In the present study, we demonstrated that piperine regulated mitochondria respiration via AMPK-related signal pathways in the skeletal system. Collectively, these facts suggest that AMPK may be an excellent research target for mitochondria both in skeletal cell and in adipocytes, but its molecular mechanism is still unclear. Further study should be focused on therapeutically usefulness of AMPK.

In summary, this study shows that piperine regulates glucose uptake by inducing the lactate-AMPK-p38 MAPK pathways and also causes a mitochondrial respiration regulation via UCP1 induction in skeletal muscle C2C12 cells. BAT is important for thermogenesis and UCP1 is necessary to mediate thermogenesis. UCP1 induction in BAT promotes energy expenditure and protects from obesity. The key effect of diabetes drug, metformin, is to decrease hepatic glucose production through OCR reduction via inhibition of the mitochondrial respiratory-chain complex 1. In the present study, piperine induces UCP1 expression and at the same time, reduces OCR. Thus, we demonstrate that piperine has strong potential to be used as a novel therapeutic agent to treat metabolic disorders such as type 2 diabetes and obesity.

## Methods

### Reagents

Piperine, metformin, Sodium L-lactate, Guanosine 5′-diphosphate sodium salt (GDP, UCP1 inhibitor) and monoclonal anti-β-actin antibody were purchased from Sigma Chemical Company (St. Louis, MO, USA). Compound C (AMPK inhibitor) was provided by Merck (Rahway, NJ, USA). SB203580 (p38 MAPK inhibitor) and monoclonal antibody against UCP1 were purchased from Abcam (Cambridge, MA, USA). Monoclonal antibodies against phosphorylated AMPKα, phosphorylated p38 MAPK, phosphorylated TBC1D4, ACC, p38 MAPK, TBC1D4 and Glut4 and polyclonal antibodies against AMPKα and phosphorylated ACC were purchased from Cell Signaling Technology (Danvers, MA, USA). Monoclonal anti-c-Myc antibody was acquired from Santa Cruz Biotechnology (Dallas, TX, USA). Hybond electrochemiluminescence (ECL) nitrocellulose membranes were obtained from GE Healthcare (Little Chalfont, Buckinghamshire, UK).

### Cell culture

Mouse C2C12 myoblasts, rat L6 myoblasts and 3T3-L1 pre-adipocytes were maintained in DMEM supplemented with 10% heat-inactivated FBS, 100 U/mL penicillin, and 100 μg/mL streptomycin at 37 °C in a humidified atmosphere of 5% CO_2_. Rat L6 myoblasts were seeded in 12-well plates at a density of 2 × 10^4^ cells/ml for differentiation into myotubes that were used in glucose uptake studies. After 24 h (at >80% confluence), the medium was replaced by DMEM containing 2% (v/v) FBS. Thereafter, the medium was replaced after 2, 4, and 6 days of culture. Experiments were initiated after 7 days when myotube differentiation was complete.

### MTT assay

Cell viability was assessed using a CellTiter 96^®^ Non-Radioactive Cell Proliferation Assay (MTT) kit (Promega, Madison, WI, USA), based on the reduction of MTT into formazan dye by the action of mitochondrial enzymes. Briefly, C2C12 cells were seeded in 96 well plates at 1 × 10^4^ cells/well and incubated overnight at 37 °C with 5% CO_2_. The cells were treated with indicated concentrations (0, 1, 10 and 30 μM) of piperine for 3 hours. The absorbance of each well was measured at 570 nm.

### NMR analysis and data pre-processing

Polar metabolites were extracted from cells with a solvent composed of methanol, distilled water, and chloroform. ^1^H-NMR spectra were measured using an 800-MHz NMR instrument. A NOESYPRESAT pulse sequence was applied to suppress the residual water signal. For each sample, 256 transients were collected into 64,000 data points using a spectral width of 16393.4 Hz with a relaxation delay of 4.0 s and an acquisition time of 2.00 s. All NMR spectra were phased and baseline-corrected using the Chenomx NMR suite version 6.0 (Chenomx Inc., Edmonton, Alberta, Canada). ^1^H-NMR spectra were segmented into 0.005-ppm bins. Spectral data were normalized to the total spectral area. Data files were imported into MATLAB (R2006a; Mathworks, Inc., Natick, MA, USA), and all spectra were aligned using the correlation optimized warping (COW) method^55^.

### Mitochondrial oxygen consumption rate

Cell respiration was measured using a Seahorse XF24 Analyzer (North Billerica, MA, USA). C2C12 cells were seeded in an XF-24-well cell culture microplate at 2 × 10^4^ cells/well and incubated overnight at 37 °C with 5% CO_2_. The cells were treated with 30 μM piperine or 10 mM metformin for 24 h, and the medium was replaced with unbuffered DMEM supplemented with 25 mM glucose, 4 mM L-glutamin, and 1 mM pyruvate (Sigma). Each cycle included 3 min of mixing, 2 min waiting and measurement over 2 min. Three measurements were obtained at baseline and following injection of 1 μM oligomycin, 1 μM FCCP and 0.5 μM rotenone/antimycin A. Mitochondrial respiration was quantified according to the oxygen consumption rate.

### Western blot analysis

The cells were grown in six-well plates. After achieving 60–70% confluence, the cells were serum starved for 24 h before treatment with selected agents at 37 °C. The cells were then treated with 30 μM piperine for 3 h. After the treatment, the medium was aspirated. The cells were washed twice with ice-cold phosphate-buffered saline (PBS) and were lysed in 100 μL lysis buffer (0.5% deoxycholate, 0.1% sodium dodecyl sulfate [SDS], 1% Nonidet P-40, 150 mM NaCl, and 50 mM Tris-HCl [pH 8.0]) containing proteinase inhibitors (0.5 μM aprotinin, 1 μM phenylmethylsulfonyl fluoride, and 1 μM leupeptin; Sigma). The supernatants were briefly sonicated, centrifuged for 20 min, and then heated for 10 min at 95 °C. After separating on a 10% SDS-polyacrylamide gel, proteins were transferred onto polyvinylidene difluoride membranes. The membranes were incubated at 4 °C overnight with primary antibodies, after which they were washed six times with Tris-buffered saline containing 0.1% Tween-20. The membranes were then incubated with horseradish peroxidase (HRP)-conjugated secondary antibodies for 1 h at room temperature. Anti-β-actin antibody was used to normalize protein loading. The blots were visualized using an ECL solution (Thermo Fisher Scientific, Foster City, CA, USA). Quantitation was performed by densitometry using Image J.

### Uptake of 2-deoxy-d(H^3^)-glucose

Glucose uptake was determined by measuring the uptake of 2-deoxy-d(H^3^)-glucose (2-DG) by differentiated L6 myotubes. The cells were rinsed twice with warm PBS (37 °C) and then starved in serum-free DMEM for 3 h. After piperine treatment, the cells were incubated in KRB (20 mM HEPES [pH 7.4], 130 mM NaCl, 1.4 mM KCl, 1 mM CaCl_2_, 1.2 mM MgSO_4_, and 1.2 mM KH_2_PO_4_) containing 0.5 μCi 2-DG at 37 °C for 15 min. The reaction was terminated by placing the plates on ice and by washing the cells twice with ice-cold PBS. The cells were then lysed in 0.5 N NaOH, and 400 μL cell lysate was mixed with 3.5 ml scintillation cocktail. Radioactivity was measured by scintillation counting.

### RNA extraction

Total RNA was extracted from 1 × 10^6^ cells/ml using an RNeasy Mini Kit (Qiagen, Valencia, CA, USA) according to the manufacturer’s protocol. RNA concentration and quality were immediately determined using a Nanodrop 2000 (Thermo Fisher Scientific), and aliquots of the total RNA were stored at −80 °C until further use.

### RT-PCR

Total RNA (500 ng) was reverse transcribed using AMV Reverse Transcriptase (Promega). The cDNA was amplified using a GeneAmp PCR System 9700 (Applied Biosystems, Foster City, CA, USA) and then heated to 95 °C for 5 min to inactivate the reverse transcriptase. PCR was performed using 30 cycles of denaturation at 94 °C for 30 s, annealing at 55 °C for 30 s, and amplification at 72 °C for 60 s followed by final elongation at 72 °C for 10 min. The number of PCR cycles was optimized to ensure that the amplification was performed in an exponential phase. Next, 6 μl PCR products was analyzed by 1% agarose gel electrophoresis. The bands obtained were stained with ethidium bromide and visualized under ultraviolet light. Band intensities were quantified using UVP BioDoc.it imaging system (Upland, CA, USA). The PCR was performed using the following primers: Glut4 sense (5′-TTG GAG AGA GAG CGT CCA AT-3′) and antisense (5′-CTC AAA GAA GGC CAC AAA GC-3′) and β-actin sense (5′-CAG GAG GAG CAA TGA TCT TGA-3′) and antisense (5′-ACT ACC TCA TGA AGA TCC TCA-3′). Each experiment was repeated three times.

### RT-qPCR

Total RNA (50 ng) was used as template to synthesize cDNA using the GoTaq^®^ 1-Step RT-qPCR System according to the manufacturer’s instructions (Promega). Reactions were carried out with SYBR green for 40 cycles of denaturation at 95 °C for 10 s, annealing at 60 °C for 30 s, and extension at 72 °C for 30 s using the StepOnePlus Real-Time PCR System (Applied Biosystems). The qPCR was performed using the following primers: Glut4 sense (5′-GAT TCT GCT GCC CTT CTG TC-3′) and antisense (5′-ATT GGA CGC TCT CTC TCC AA-3′), UCP1 sense (5′-GGG ACC TAC AAT GCT TAC AGA GTT-3′) and antisense (5′-TCA TCT GCC AGT ATT TTG TTG TTT-3′), UCP2 sense (5′-GCG TTC TGG GTA CCA TCC TA-3′) and antisense (5′-GCT CTG AGC CCT TGG TGT AG-3′), UCP3 sense (5′-ATG AGT TTT GCC TCC ATT CG-3′) and antisense (5′-GGC GTA TCA TGG CTT GAA AT-3′), FGF21 sense (5′-GAT CAG GGA GGA TGG AAC AGT-3′) and antisense (5′-TCA AAG TGA GGC GAT CCA TAG-3′), Tmem26 sense (5′-CTC TTG CTG GTC CTG GAG AC-3′) and antisense (5′-GGG TGC TGC AAT ACT GGT TT-3′), PGC-1α sense (5′-ATG TGT CGC CTT CTT GCT CT-3′) and antisense (5′-ATC TAC TGC CTG GGG ACC TT-3′), and β-actin sense (5′-TGT TAC CAA CTG GGA CGA CA-3′) and antisense (5′-GGG GTG TTG AAG GTC TCA AA-3′). The experiment was performed on three independent biological replicates. Gene expression was normalized to the mRNA expression level of β-actin as an endogenous control, and fold changes were calculated between treatment and negative-treatment control samples.

### Mitochondria isolation

C2C12 cells were seeded in 100 mm cell culture dish at 1 × 10^6^ cells/well and incubated overnight at 37 °C with 5% CO_2_. The cells were pre-treated with 1 mM GDP for 30 min and treated with 30 μM piperine for 3 h and then washed the dish with cold PBS. Mitochondria isolation was performed using Mitochondria Isolation Kit for Cultured Cells (Thermo Fisher Scientific), according to the manufacturer’s protocol. The isolated mitochondria were stored at −80 °C until the Western blot analysis.

### Silencing of genes encoding AMPKα2 and p38 MAPK

Cells were seeded in six-well plates, cultured for 24 h to 70% confluence, and then transiently transfected with siRNAs against genes encoding AMPKα2 (L-040809, Dharmacon, GE Healthcare) and p38 MAPK (L-040125, Dharmacon) using Lipofectamine 2000 (Invitrogen, Life Technologies, Carlsbad, CA, USA), according to the manufacturer’s protocol. For transfection, 5 μL siRNAs and 5 μL Lipofectamine 2000 were diluted using 95 μL reduced serum medium (Opti-MEM, Invitrogen, Life Technologies) and mixed. The mixture was incubated for 30 min at room temperature and then added dropwise to each culture well containing 800 μL Opti-MEM (final siRNA concentration, 100 nM). The medium was replaced with fresh complete medium after 6 h of transfection.

### Myc-Glut4 translocation assay

Cell surface expression of Myc-Glut4 was quantified by performing an antibody-coupled colorimetric absorbance assay as described previously^56^. After piperine stimulation, L6 myotubes that stably expressed Myc-Glut4 were incubated with polyclonal anti-Myc antibody (1:1000) for 60 min, fixed with 4% paraformaldehyde in PBS for 10 min, and incubated with HRP-conjugated goat anti-rabbit antibody (1:1000) for 1 h. The cells were then washed six times with PBS and incubated in 1 ml *o*-phenylenediamine (0.4 mg/mL) for 30 min. Absorbance of the supernatant was measured at 492 nm.

### Preparation of primary myoblasts

Primary myoblasts were isolated from the forelimbs and hindlimbs of three or four 5-day-old littermates^57^. The muscles were dissected and minced, disaggregated enzymatically in 4 ml PBS containing 1.5 U/mL dispase II and 1.4 U/mL collagenase D (Roche, Grenzacherstrasse, Basel, Switzerland), and triturated with a 10-ml pipette every 5 min for 20 min at 37 °C. The cells were filtered through a 70-μm mesh (BD Bioscience, San Jose, CA, USA) and centrifuged at 1000 × *g* for 5 min. The cell pellet was dissociated in 10 mL F10 medium (Invitrogen, Life Technologies) supplemented with 10 ng/mL basic fibroblast growth factor (PeproTech; Rocky Hill, NJ, USA) and 10% cosmic calf serum (GE Healthcare). Finally, the cells were pre-plated twice on non-collagen coated plates for 1 h to deplete fibroblasts, which generally adhere faster than myoblasts. For differentiation, the primary myoblasts obtained were cultured to 75% confluence in DMEM containing 100 U/mL penicillin, 100 μg/mL streptomycin, and 5% horse serum (Invitrogen, Life Technologies).

### ATP and lactate measurements

Intracellular ATP levels were quantified using an ATP assay kit (Abcam, Cambridge Science Park, Cambridge, UK). Lactate concentrations in C2C12 cells supernatants were measured using a Lactate colorimetric assay kit (BioVision, Milpitas, CA, USA).

### Data analysis

Multivariate statistical analyses of NMR data were performed with Pareto scaling using SIMCA-P+ software, version 12.0 (Umetrics, Umeå, Sweden). All changes in metabolite levels, including isoleucine, leucine, valine, AMP, ADP, ATP, lactate, fumarate, and malate, were assessed by Student’s *t-*test using GraphPad Prism (version 5 for Windows; GraphPad Software). Data are presented as mean ± standard deviation (SD) of individual experiments. All experiments were performed with at least three independent replicates. The difference between mean values was considered statistically significant at p < 0.05.

## Additional Information

**How to cite this article**: Kim, N. *et al*. Piperine regulates UCP1 through the AMPK pathway by generating intracellular lactate production in muscle cells. *Sci. Rep.* **7**, 41066; doi: 10.1038/srep41066 (2017).

**Publisher's note:** Springer Nature remains neutral with regard to jurisdictional claims in published maps and institutional affiliations.
